# Assessing Grain Quality Changes in White and Black Rice under Water Deficit

**DOI:** 10.3390/plants12244091

**Published:** 2023-12-07

**Authors:** Aloysha Brunet-Loredo, María Dolores López-Belchí, Karla Cordero-Lara, Felipe Noriega, Ricardo A. Cabeza, Susana Fischer, Paula Careaga, Miguel Garriga

**Affiliations:** 1 Department of Plant Production, Faculty of Agronomy, University of Concepcion, Avenida Vicente Mendez, 595, Chillán 3780000, Chile; abrunet@udec.cl (A.B.-L.); mlopezb@udec.cl (M.D.L.-B.); fnoriega@udec.cl (F.N.); sfischer@udec.cl (S.F.); agro.paulacr@gmail.com (P.C.); 2 Institute of Agricultural Research, Regional Research Center Quilamapu, Avenida Vicente Mendez, 515, Chillán 3780000, Chile; kcordero@inia.cl; 3 Plant Nutrition Laboratory, Department of Crop Sciences, Faculty of Agricultural Sciences, University of Talca, Avenida Lircay s/n, Talca 3460000, Chile; rcabeza@utalca.cl

**Keywords:** bioactive compounds, phenolic compounds, antioxidant activity, mineral composition, water stress, functional food

## Abstract

Rice is an essential diet component for a significant portion of the population worldwide. Due to the high water demand associated with rice production, improving water use efficiency and grain quality is critical to increasing the sustainability of the crop. This species includes rice varieties with diverse pigmentation patterns. Grain quality, including industrial, nutritional, and functional quality traits, of two black rice genotypes and a commercial white rice cultivar were evaluated in different locations and under different water regimes. Flooding produced higher grain weight compared to alternate wetting and drying irrigation. A high correlation was found between grain color, total phenolic content (TPC), and antioxidant activity. The black rice genotypes showed higher TPC levels and antioxidant capacity, mainly due to higher levels of cyanidin 3-*O*-glucoside. The phenolic profile varied between whole and polished grains, while mineral composition was influenced by location and irrigation regime. In turn, the environment influenced grain quality in terms of industrial and nutritional characteristics, with significant differences in quality between whole and polished grains. This study provides valuable information on the genotype–environment relationship in rice and its effect on grain quality, which could contribute to selecting genotypes for an appropriate environment.

## 1. Introduction

Cultivated rice (*Oryza sativa* L.) plays a crucial role as a staple food globally. In fact, approximately 25% of calorie and protein intake of the world’s population comes from rice [1], representing up to 75% of the calories consumed by Asian countries [2]. In Chile, rice is grown in the region located between 35° and 36° S, which is the southernmost rice production area in the world [3]. Locally, yield reached 4.86 t ha^−1^ in an area of 20.7 thousand ha in the 2021/2022 season [4]. Due to the peculiar climatic conditions of the Chilean rice region, locally adapted cultivars are needed.

White rice *O. sativa* is the most widely consumed species worldwide, but in nature, there is a wide range of pigmented rice, ranging in color from brown to red to deep purple-black, which belongs to this species [5]. Black rice, predominantly cultivated in Asia, is recognized as “forbidden rice” or “king’s rice” in China, its leading producer. It is also cultivated in neighboring countries, such as Sri Lanka, India, the Philippines, and Thailand [5,6]. The deep purple hue of the grain is due to the accumulation of phenolic compounds, mainly anthocyanins, in the pericarp [7,8]. At a global scale, pigmented rice accounts for only 0.1% of the total rice production, which is attributed to poor awareness of this rice variety, relatively low yield, and consumer preferences for polished white rice [9].

Pigmented rice contains several bioactive and nutritional compounds, such as anthocyanins, flavanones, phenolic acids, vitamins, fatty acids, and minerals [10]. Unlike white rice, black rice genotypes are rich in anthocyanins [11,12], mainly cyanidin 3-*O*-glucoside and peonidin 3-*O*-glucosid. However, other compounds, such as pelargonidin 3-*O*-glucoside and cyanidin 3-*O*-arabinoside, have also been identified. Compared to white rice, pigmented rice grains have higher contents of phenolic acids [12], including ferulic acid, *p*-coumaric acid, and vanillic acid [12,13], as well as higher levels of γ-oryzanol [14] and carotenoids, namely lutein and zeaxanthin [11,15]. Furthermore, black rice has been reported to offer greater nutritional benefits compared to white rice, with higher levels of protein, minerals, and amino acids. However, the extent of these benefits depends on environmental factors and production management [5,8]. Black rice contains niacin and eighteen amino acids, and it is also a reliable source of thiamine and fatty acids, with a high concentration of essential elements (Fe, Zn, Ca, K, Mg, and Mn) [5,13,15]. Comparative studies have also shown that black rice contains more dietary fiber (21–52%) and total protein content (7–24%), and less total soluble carbohydrates (29–35%) than white rice [6,15].

Several studies have documented the nutraceutical properties of black rice and underscored its potential health benefits, including the prevention of cardiovascular diseases, obesity, type II diabetes, and different types of cancer [5,10,16,17]. These attributes, along with the substantial potential of black rice for functional food production [6,10,16], have resulted in an increasing consumer interest and demand for black rice in European, North American, and Australian markets [5]. In Chile, there is also great interest in this type of rice, and the Black Rice Breeding Program (BRBP) was recently implemented at the Institute of Agricultural Research (INIA) in order to develop cultivars suitable for local conditions.

Rice cultivation in Chile is predominantly based on flooding methods, posing challenges to sustainability and productivity derived from increasing water scarcity, mainly attributed to climate change [4,18]. Water deficit affects plant growth, development, and yield [19]. In this sense, several studies have indicated that rice quality traits, such as milling rate [20,21], protein and amylose content [22], antioxidant content [23,24], mineral nutrients [25,26], and culinary quality [4,27], are also affected by water stress. Most of these studies were conducted on traditional white rice.

Considering the current scenario of water scarcity and the effects of water deficit on rice cultivation, the recently established BRBP aims at developing cultivars with increased performance and grain quality under water-limiting conditions. The objective of this study is to compare the grain quality and nutritional traits of two black rice genotypes and a commercial white rice cultivar developed by INIA under contrasting water conditions.

## 2. Results

### 2.1. Appearance and Sensorial Quality Traits

The commercial white rice cultivar Zafiro-INIA showed the highest thousand-grain weight in both paddy and polished rice, differing significantly from Quila 279101 and Quila 292008 (Table 1). The interaction of genotype (G) × location (L) × water regime (W) was statistically significant for thousand-grain weight in paddy rice, while the interaction of L × W was statistically significant in polished rice. All the interactions were significant for grain length and length/width ratio except for location and water regime. Meanwhile, only G × W was significant for grain width.

Differences between locations were detected. The highest grain weight was obtained in Parral, with increases of 4.9% and 2.1% in paddy and polished rice, respectively. Differences in grain weight were also observed for water regime, being higher in the flooded plants and reaching 6.1% and 2.7% in paddy and polished rice, respectively.

Whole-grain (WG) yield showed differences between genotypes, locations, and water regimes (Table 2). No significant interactions between factors were observed in WG except for G × W. Regarding genotypes, the highest WG value was obtained in Zafiro-INIA, being 27% higher than that of Quila 279101, but no statistical differences were found between Quila 292008 and Zafiro-INIA. San Carlos yielded 11.5% more WG than Parral, while this parameter was 41.2% higher under AWD irrigation compared to the yield obtained under flooded conditions.

As expected, chalkiness and transparency were statistically different between white and black rice. Similarly, differences were found between black rice genotypes, with Quila 292008 having higher values for chalkiness and transparency compared to those of Quila 292008 (Table 2). There were no differences in these traits between cultivation sites. As for water regimes, grains grown under flooded conditions showed 8.8% higher chalkiness. In addition, none of the interactions between factors were significant for chalkiness and transparency.

There were significant differences in the presence of white belly (WB) in the grain between genotypes and water regimes but not between locations (Table 2). The highest WB values were observed in black rice genotypes, with Quila 279101 and Quila 292008 being 2.2- and 3.3-fold higher, respectively, compared to Zafiro-INIA. Under flooded conditions, WB was more than twice as high as in AWD. No differences in WB were detected between factors except for G × W. The same was true for WB; alkaline dispersion (ADD) showed significant differences between genotypes and water regimes, but no differences were found between growing sites. The highest ADD value was observed in Quila 279101, which was 10.8% higher than in Zafiro-INIA. For flooding, ADD was 6.2% higher than in AWD.

### 2.2. Nutritional Quality Traits

Phenolic concentration was higher in pigmented rice grains and, to a greater extent, in whole grains (Tables S3 and S4). Both black rice genotypes showed a mean concentration of 2.57 mg g^−^^1^ in polished grains, although the concentration in Quila 279101 was 37.1% higher than in Quila 292008. On the other hand, phenolic compounds in whole Zafiro-INIA grains averaged 0.105 mg g^−^^1^, which was 24.5 times lower than levels in pigmented grains (Table S3). Regarding locations, pigmented genotypes grown in Parral averaged 2.6 times more compounds than those grown in San Carlos, recording concentrations of 3727 mg g^−^^1^ and 1418 mg g^−^^1^, respectively. However, the concentration of compounds in whole black grains showed less variation due to water management, with 2360 and 2785 mg g^−^^1^ under flooding and AWD regimes, respectively. In black rice grains, the highest concentrations of compounds corresponded to cyanidin 3-*O*-glucoside followed by apigenin derivative and quercetin, accounting for 78.72%, 5.32%, and 2.90% of the sum of phenolic compounds, respectively. In white rice grains, neither cyanidin 3-*O*-glucoside nor quercetin was detected, while the most predominant compound was chlorogenic acid, accounting for 39.37% of the sum of phenolic compounds (Table S3).

The total mean phenolic concentration in polished pigmented grains was 0.76 mg g^−^^1^ (Table S4), which was 3.4 times lower than that in whole black grains (Table S3). Polished Quila 292008 recorded the highest concentration, regardless of location and water regime, with a mean of 1.24 mg g^−^^1^, which was 4.3 times higher than that of polished Quila 279101. On the other hand, polished grains from Zafiro-INIA showed a mean value of 0.04 mg g^−^^1^, which was 20.6 times lower than that of pigmented grains. In addition, location had an impact on whole grains; the mean value of pigmented rice from San Carlos was 2-fold that of Parral. For water regimes, polished black grains showed the same trend as whole black rice, with a minor difference in phenolic concentration between flooding and AWD irrigation of 0.72 and 0.81 mg g^−^^1^, respectively (Table S4). Similar to what was observed in whole grain, the main compounds in polished black grains were cyanidin 3-*O*-glucoside and apigenin derivative, which accounted for 76.85% and 6.52% of the total concentration, respectively. In white rice grains, caffeic, vanillic, and chlorogenic acids recorded the highest values, accounting for 82.58% of the total phenolic content (TPC) (Table S4).

**Figure 1:**
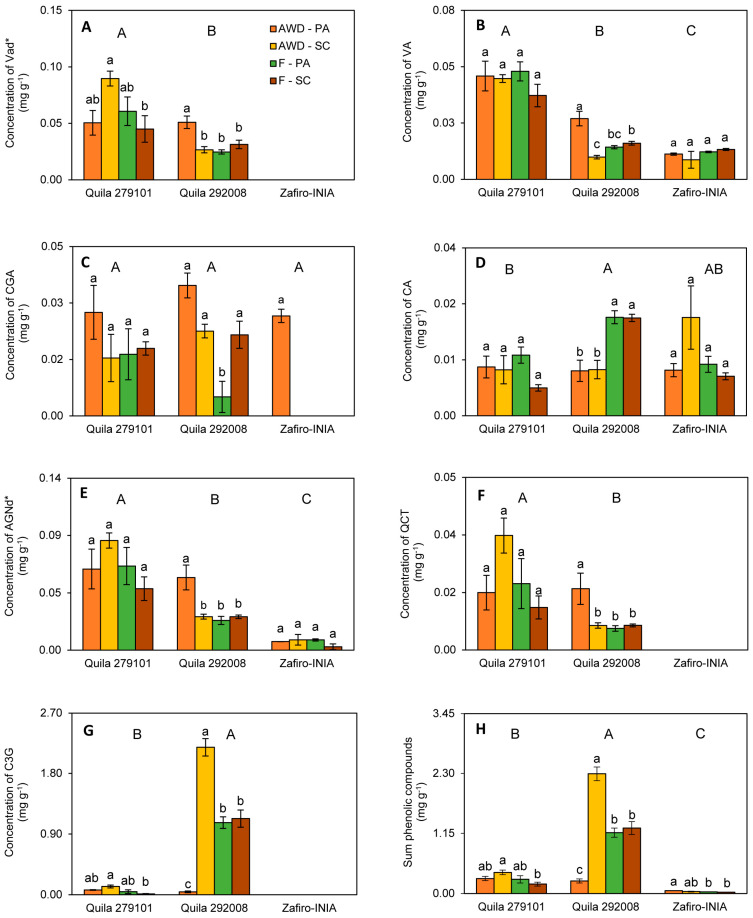
Concentration of phenolic compounds in polished grains of three rice genotypes (Quila 279101, Quila 292008, and Zafiro-INIA) grown in different environments (AWD-PA, AWD-SC, F-PA, and F-SC). (**A**) vanillic acid derivative, (**B**) vanillic acid, (**C**) chlorogenic acid, (**D**) caffeic acid, (**E**) apigenin derivative, (**F**) quercetin, (**G**) cyanidin 3-*O*-glucoside, and (**H**) Sum of phenolic compounds. AWD: alternate wetting and drying, F: flooding. PA: Parral, SC: San Carlos. Data are mean values ± standard error (n = 3). Means with different letters are statistically different according to the LSD Fisher test (*p* ≤ 0.05). Capital letters indicate differences among genotypes. Lowercase letters indicate differences between environments for each genotype.

When water regime and location were analyzed as a combined environment (Figure 1), statistical differences were observed between genotypes for all phenolic compounds except for chlorogenic acid. Regarding the effect of the environment on the TPC, the most significant differences were found in the concentration of Vad*, VA, AGNd*, and QCT in Quila 292008 (Figure 1A,B,E,F). Few differences were found between environments for the other two genotypes. However, Quila 279101 showed higher concentrations in most compounds except for cyanidin 3-O-glycoside; Quila 292008 showed a higher concentration of this compound, especially in the AWD-SC environment (Figure 1G). Similarly, the latter environment affected the total concentration of phenolic compounds (Figure 1H). The Zafiro-INIA genotype showed the presence of VA, CA, and AGNd* concentrations in all the environments, while only CGA concentration was detected in AWD-PA (Figure 1C).

The TPC and antioxidant capacity of polished grain extracts are shown in Table 3. Significant differences were observed in the G × L × W interaction in TPC, ORAC, and DPPH. All of them showed similar differences between genotypes, which were significantly higher in black rice compared to white rice. The highest values were observed in Quila 279101, being 3.8, 3.6, and 8.7 times higher for TPC, ORAC, and DPPH, respectively, with respect to Zafiro-INIA. No differences were observed between location and water regimes.

**Figure 2:**
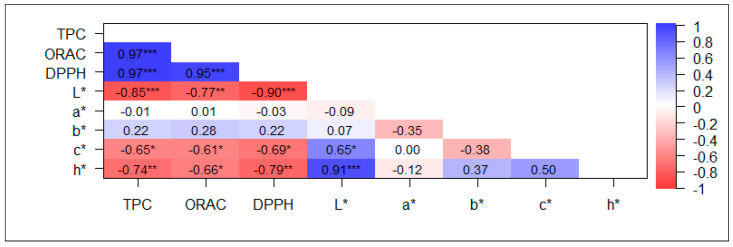
Pearson correlation analysis between total phenolic content (TPC), antioxidant capacity (ORAC and DPPH), and color parameters (L*, a*, b*, c*, and h*) in whole rice grain. * *p* < 0.05, ** *p* < 0.01, and *** *p* < 0.001. L*: luminosity, a*: a measure of redness, b*: a measure of yellowness, c*: chroma, saturation, h*: hue angle. n = 12.

**Figure 3:**
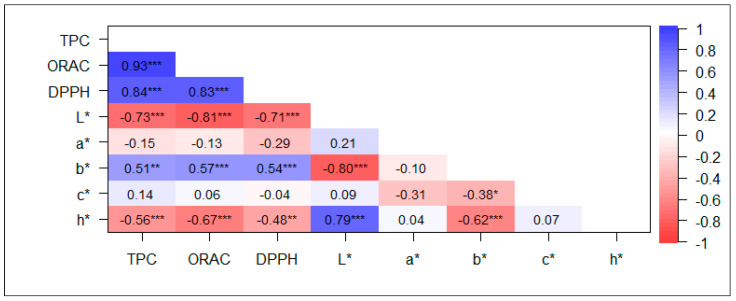
Pearson correlation analysis between total phenolic content (TPC), antioxidant capacity (ORAC and DPPH), and color parameters (L*, a*, b*, c*, and h*) in polished rice grain. * *p* < 0.05, ** *p* < 0.01, and *** *p* < 0.001. L*: luminosity, a*: a measure of redness, b*: a measure of yellowness, c*: chroma, saturation, h*: hue angle. n = 36.

Correlations between antioxidant capacity and TPC of whole and polished rice grains were high and positive (Figure 2 and Figure 3), particularly in whole grains, reaching values higher than 0.95. Among the color parameters, L* showed the highest correlations with TPC, ORAC, and DPPH, ranging between −0.769 and −0.898 and between −0.705 and −0.805 in whole and polished grains, respectively. The c* and h* parameters showed moderately high correlations with antioxidant capacity and TPC in whole grains, ranging from −0.611 to −0.793; h* behaved similarly in polished grains but to a lesser extent, being in the range of −0.480 and −0.665.

### 2.3. Relative Mineral Composition

**Figure 4:**
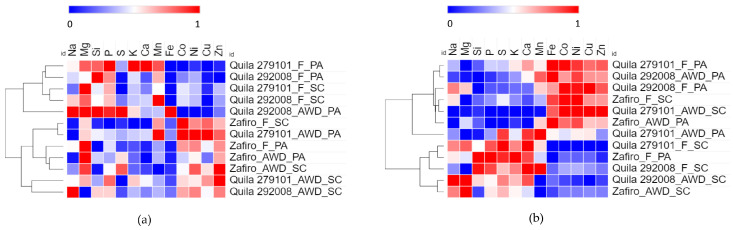
Heat map and cluster analysis of mineral relative content in rice with different genotypes (Quila 279101, Quila 292008, and Zafiro-INIA), locations (San Carlos, SC, and Parral, PA), and irrigation regimes (flooding, F, and alternate wetting and drying, AWD). (**a**) Polished grain (n = 36) and (**b**) whole grain (n = 12). The mineral relative content (KeV) data were normalized to the 0–1 scale for the analysis.

The mineral composition in processed and whole grains was subjected to a heat map and cluster analysis, and the diverse patterns observed reveal distinct element distributions among different genotypes and environments (Figure 4). Segregation based on water regime and grain type was observed in polished grains (Figure 4a). One main cluster included black rice genotypes cultivated under flooded conditions and Quila 292008-AWD-PA. All genotypes grown under AWD irrigation, except for Quila 292008-AWD-PA, and all Zafiro-INIA were included in the second cluster, regardless of irrigation regime and location, indicating differences regarding water conditions and rice types. Compared to AWD rice, flooded rice had lower relative contents of Co, Ni, Cu, and Zn, whereas levels of Mn, P, and Si tended to increase. On the other hand, the levels of relative Fe remained persistently low despite the variations observed in genotypes, water regimes, and locations. Conversely, Mg tended to reach a high relative content.

The first main cluster in whole grains comprised all genotypes cultivated in Parral except for Zafiro-F-PA. This cluster included Zafiro-F-SC and Quila 279101-AWD-SC. The second cluster grouped the remaining genotypes from San Carlos and Zafiro-F-PA, indicating potential environmental variations due to locations (Figure 4b). The first cluster showed increased levels of Fe, Co, Ni, Cu, and Zn compared to those of Ca, K, S, P, and Si. The second group, mostly consisting of grain from San Carlos, exhibited an opposing trend. Unlike polished grains, no separation was observed between pigmented and white grain genotypes.

Significant differences were observed in the relative contents of P, K, Ca, and Mn between Quila 279101 and Quila 292008 as well as in Zafiro-INIA. The Na content in Quila 292008 significantly differed from Zafiro-INIA, but there were no differences with respect to Quila 279101. In addition, no differences were found in terms of relative mineral contents for the growing site or water regime except for levels of Zn, as higher values were observed under AWD irrigation (Table S1).

### 2.4. Principal Component Analysis (PCA) of Grain Quality Traits

**Figure 5:**
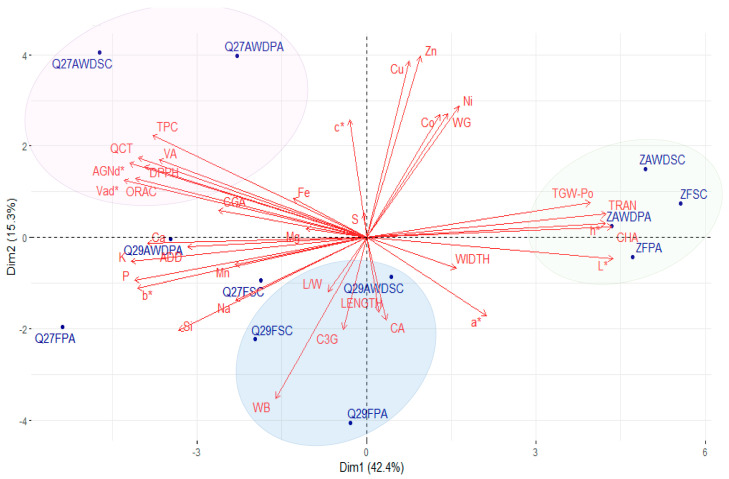
Principal component analysis (PCA) biplot of quality traits of polished rice grain of three genotypes (Quila 279101, Q27; Quila 292008, Q29; and Zafiro, Z) cultivated in two locations (San Carlos, SC; and Parral, PA) under two irrigation regimes (flooding, F; and alternate wetting and drying, AWD).

**Figure 6:**
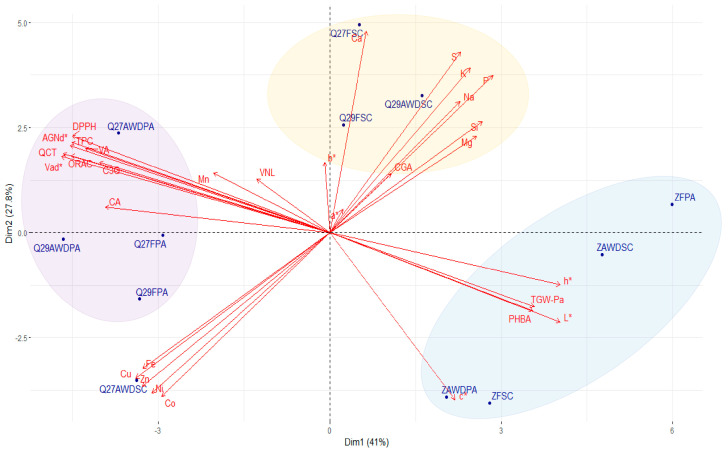
Principal component analysis (PCA) biplot of quality traits of whole rice grain of three genotypes (Quila 279101, Q27; Quila 292008, Q29; and Zafiro, Z) cultivated in two locations (San Carlos, SC; and Parral, PA) under two irrigation regimes (flooding, F; and alternate wetting and drying, AWD).

A PCA was developed to establish relationships between whole and polished grain quality traits with genotype, irrigation regime, and location (Figure 5 and Figure 6). In general, an apparent separation was observed between the black genotypes and the white rice cultivar and, to a greater extent, in polished grains. The separation was mainly due to significant differences in color and higher grain weight of the white rice. The PCA performed for the polished grains explained 57.7% of the variance in the data (Figure 5). All Zafiro-INIA samples clustered closely, regardless of water regime and location. This clustering was determined by TGW-Po and grain appearance traits, such as TRAN, CHA, h*, and L*, and to a lesser extent by WIDTH, a*, WG, and relative mineral content of Zn, Ni, Cu, and Co. All black rice genotypes were in the opposite quadrants of the PCA except for Quila 292008-AWD-SC. The separation of pigmented rice genotypes was associated with the concentration of phenolic compounds such as QCT, CGA, VA, Vad*, AGNd*, AGNd*, and AGNd*; biological activity (TPC, ORAC, DPPH); color parameters, i.e., b*; relative contents of K, P, Ca, and Si; and ADD.

In contrast to white grains, pigmented grains showed a much broader distribution in the PCA, with no clear grouping by genotype, water regime, or location (Figure 5). However, Quila 279101 under AWD irrigation was separated from the rest, irrespective of the growing site, which was mainly determined by the biological activity traits TPC, DPPH, and ORAC and the concentration of metabolites such as QCT, AGNd*, Vad*, and VA. Quila 279101 grown under flooded conditions clustered in the lower quadrant. On the other hand, Quila 292008 tended to cluster, regardless of water regime and location, which was influenced by the presence of WB and, to a lesser extent, by C3G and CA concentration, LENGTH, and L/W ratio.

The PCA for whole grains explained 68.8% of the data variance of the first two components (Figure 6). The traits Vad*, QCT, AGNd*, ORAC, TPC, DPPH, VA, h*, L*, C3G, CA, TGW-Pa, PHBA, and Cu had the highest contribution to the first two components. As for polished grains, Zafiro-INIA clustered but less tightly, and interestingly, no association with water regime or locations was observed. Likewise, grain weight (TGW-Pa), p-hydroxybenzoic acid (PHBA), and color parameters h* and L* played a significant role in this clustering. Black rice genotypes exhibited a clear tendency to cluster by location, regardless of genotype and water regime, except for Q279101-AWD-SC, which was separated from the San Carlos group. This group was determined by the relative content of minerals such as Ca, S, K, Na, P, Si, and Mg. In contrast, the Parral group was associated with the concentration of phenolic compounds (QCT, VAd*, AGNd*, VA, and C3G) and biological activity (TPC, ORAC, and DPPH).

## 3. Discussion

### 3.1. Appearance and Sensorial Features

Grain quality is critical for breeders, producers, and consumers [20,28]. Traditional rice quality parameters encompass aspects related to grain appearance, including weight, size (length and width), shape (length/width ratio), whiteness, and transparency [4,28,29]. On the other hand, culinary and sensory quality parameters include factors such as gelatinization temperature, gel consistency, and cooking method [4,27], which collectively influence consumer preferences [28].

There is a growing interest in black rice due to its high nutritional value and functional quality [5,6,8,10,14]. However, farmers in many countries are unwilling to grow this species because it has a lower yield potential compared to white rice [5]. This is reflected in the significant differences in grain weight between Zafiro-INIA and black rice genotypes (Table 1). In addition, rice grain formation and filling are negatively affected by water-deficit conditions [26,30] such as AWDI irrigation, resulting in decreased grain weight. The TGW-Pa values obtained under flooded and AWD conditions exceeded those reported for pigmented rice by Rungrat and Poothab [30] by 11.2% and 5.5%, respectively. Furthermore, a two-year study conducted by Wang et al. [31] evaluated two japonica rice varieties under water deficit, reporting average values for TGW-Pa ranging from 23.6 to 25.2 g and from 22.9 to 24.4 g under mild and severe drought stress, respectively. These values were notably lower than those obtained in the present study.

The value of rice is related to the percentage of whole grain, since the higher the percentage of whole grain, the higher the commercial value [4]. The white rice cultivar showed the highest whole-grain percentage. However, this did not differ from that of Quila 292008. This may be associated with the highest ADD values of these genotypes (Table 2) since gelatinization promotes the recovery of cracked grain [32]. In addition, the percentage of whole grain significantly increased under AWD irrigation. Some studies have suggested that inducing soil desiccation can promote greater carbon remobilization and root expansion, thus optimizing nutrient uptake during the grain-filling stage, ultimately resulting in increased crop yields [33,34]. These processes could favor the increase in whole-grain yield under AWD conditions.

In terms of grain dimensions, all grains obtained were classified as medium-grain rice based on L/W ratio (2.1–3.0) and as long-grain rice based on the combination grain length (≥6.0 mm) and L/W ratio (2.0–3.0) [35], regardless of genotype, location, or water regime. Grain length showed no differences among genotypes, growing sites, or water regimes. Interestingly, all interactions among factors were statistically significant except for L × W. The environment affected grain width, which decreased in the AWD condition. However, this did not result in differences in the L/W ratio, which had an effect similar to length, with all the factor combinations being significant except for L × W (Table 1). The length, width, and L/W values among genotypes are similar to those reported by Noori et al. [28] in NERICA 4 and IR28 varieties. The hull, degree of filling, and endosperm development are the primary factors determining rice grain size [36]. In this sense, the effect of water deficit on grain filling may have influenced these parameters.

Chalkiness and transparency are essential quality parameters related to the appearance of rice grains. According to FAO [35], brown rice has a chalkiness value of about 20, while well-polished rice has a value of about 40. In this study, only the chalkiness level of the processed grain was determined. A chalkiness value close to optimum (36.5) was obtained in the white rice cultivar. In contrast, values were significantly lower (<16) in the black rice genotypes (Table 2), which is related to the pigment content in the grain of these genotypes. The growing site did not affect grain chalkiness, but levels decreased under water-deficit conditions. Excessive whitening in the abdominal region of grains during the grain-filling period is reported when the crop is exposed to water-deficit conditions [37]. Another study suggests that elevated night temperatures during critical grain-filling stages may influence the increase in rice chalkiness [38]. These observations highlight the complex influence of environmental and climatic factors on rice quality.

Grain transparency is associated with the appearance of a white core within the grain. Consequently, rice can be categorized into two distinct types: chalky, characterized by grains with white cores or bellies, and translucent [39]. The morphology of their endosperm distinguishes these grains. White-bellied rice exhibits numerous globular protein bodies dispersed around the starch granules, resulting in interstitial spaces. In contrast, translucent grains have tightly packed starch granules [22,39]. Due to late plantings, white-belly production has been associated with elevated temperatures and a shorter grain-filling period [4,28]. In the present study, white rice had the most translucent grain and the lowest percentage of white-bellied grains compared to black grain genotypes. In the latter, the percentage of white belly increased significantly, especially in Quila 292008. In addition, a significant G × W interaction effect was observed since rice grains produced under flooding had more than twice the percentage of white bellies compared to those produced under AWD irrigation (Table 2). As there is a genotype–environment effect of high temperature on the activation of alpha-amylase genes in starch accumulation in the grain-filling process, the stress caused by water deficit in AWD was expected to increase the percentage of white belly [40] (Figures S1 and S2), but an opposite effect was observed. However, there is scarce information on the relationship between white-belly formation and water stress.

Gelatinization temperature (GT) is a critical indicator of rice cooking quality, as it influences both cooking time and energy expenditure [27] and significantly affects the culinary quality and palatability of rice [41]. Gelatinization occurs when starch granules absorb water and lose their crystalline structure. Starch degradation can be quantified using Graham’s [42] alkaline dispersion table, which is inversely related to GT [41,43]. In this study, the black genotypes had a dispersion grade 6, corresponding to dispersed grain, neck fusion, high alkaline digestion, and low GT [43]. In contrast, Zafiro-INIA scored 5.8, being classified as fragmented grain with perfect or wide neck, intermediate alkaline digestion, and intermediate GT [43] (Table 2). In both cases, gelatinization was moderate to low [27,43]. However, it should be noted that the Zafiro-INIA grains would require a higher amount of water and a longer cooking time. These low GT values are typical of the temperate japonica rice variety [44] to which the evaluated genotypes belong. Under AWD, alkali dispersion was reduced, leading to grains with higher GT values and longer cooking times [27]. It has been reported that GT variation is explained by genotype rather than environmental factors [43,44].

### 3.2. Anthocyanins, Flavonols, and Other Phenolic Compounds

As expected, TPC was significantly higher in pigmented grains compared to the white rice cultivar. Similarly, whole rice showed higher concentrations of phenolic compounds than polished rice (Tables S2 and S3, Figures S3 and S4) because most phenolic compounds are concentrated in the aleurone layer of the grain and pericarp [5]. In this study, white and black rice genotypes tended to increase antioxidant concentrations in whole and polished grains in response to stress exposure. This response is particularly evident in the increased accumulation of anthocyanins [26,45], as observed in both black genotypes. Regardless of location and water regime, the average cyanidin 3-*O*-glucoside concentration of both black rice genotypes reached a value of 0.59 mg g^−^^1^ in polished rice, which was lower than that reported by Pereira-Caro et al. [11]. In whole rice, the mean concentration was 2.03 mg g^−^^1^, which was approximately 1.79 times lower than that reported by Colasanto et al. [46] for black rice grains. This lower anthocyanin concentration could be determined by genotypic differences and genotype–environment interaction [5], which influences the transcription of genes encoding anthocyanidin synthase [7].

In black rice, cinnamic acid is transformed into vanillic acid through an alternative pathway [10]. In this study, vanillic acid in whole-grain black rice had a mean concentration of 0.08 mg g^−^^1^, which is higher than that reported by Quan et al. [47] but lower than the values obtained by Das et al. [48]. 

Quercetin levels were notably higher in Quila 279101, in whole rice, averaging 0.08 mg g^−^^1^. This does not agree with Tyagi et al. [21], who reported a lower value for black rice. These results indicate that different black rice varieties have unique genetic profiles, resulting in variations in the expression and content of phenolic compounds [49]. It is well-known that secondary metabolite production increases under water stress [50], which corresponds to a mechanism of plant protection and acclimation in response to stress [47,50]. The accumulation of C3G, CA, VNL, and Vad* increased by 17.6, 29.6, 16.0, and 15.7%, respectively, in the whole grain of black rice due to water deficit. Similarly, in polished black grains, there were increases in QCT, CGA, AGNd*, and Vad* of 39.7, 39.1, 29.6, and 25.7%, respectively. On the other hand, whole white rice grains exhibited a 52.1% increase in AGNd* concentration, while polished grains showed increases of 36.7% in CA and 27.5% in AGNd* under AWD conditions. The increase in these phenolic compounds may directly enhance the antioxidant capacity of the plant under water-deficit conditions. Cinnamic acid and vanillin have been noted for their potent drought-tolerance properties in rice [47].

### 3.3. Total Phenolic Content (TPC) and Antioxidant Capacity (ORAC and DPPH)

The TPC analysis globally quantifies the total number of specific classes of phenolic compounds in the grain. The results of the spectrophotometric analysis were consistent with the phenolic compound identification by HPLC, revealing that TPC was significantly higher in polished black rice compared to the amount of phenolic compounds in white rice. Specifically, TPC was approximately 3.8 and 2 times higher in Quila 279101 and Quila 292008 than in Zafiro-INIA, respectively. It was described that TPC is directly associated with grain color [21], which is confirmed by our results. There was a direct correlation between the concentration of TFC and grain color, with a Quila 279101 recording the highest value of 98.37 mg GAE 100 g^−^^1^ DW due to its darker grains [21].

Although no statistically significant differences were observed between water regimes, there was a trend towards higher TPC under AWD irrigation. The TPC in the AWD treatment was 29.9% higher than that obtained in grains under flooding. This could be attributed to the high concentrations of phenolic compounds, such as C3G, CGA, CA, Vad*, or QCT in polished grains of black rice and CA and AGNd* in polished grains of the white rice cultivar, as mentioned above.

Under water-stress conditions, reactive oxygen species (ROS) accumulate in excess in plant cells, generating oxidative stress and causing DNA, protein, and membrane damage [51]. Hence, the increase in polyphenol content may represent an acclimation response since these compounds participate in scavenging excess ROS during water stress [23,52]. Through their hydroxyl groups, phenolic compounds can donate electrons, enabling them to engage in antioxidant activity [53,54]. In addition to their role in ROS scavenging, it has been suggested that phenolic compounds promote secondary cell wall thickening during water stress, enhancing resistance to oxidative damage [52,53] and contributing to improved plant acclimation under such conditions [9]. Black rice is rich in bioactive compounds, primarily anthocyanins such as cyanidin 3-*O*-glucoside, responsible for its distinctive coloration and potent antioxidant properties [5,6,8].

The ORAC and DPPH methods were used to evaluate antioxidant activity. As expected, the same effect observed in TPC was obtained with both methods. The antioxidant activity was higher in polished grains of black rice genotypes compared to that of Zafiro-INIA, ranging from 1.8 to 3.6 and 4.9 to 8.7 according to ORAC and DPPH assays, respectively. Following the same trend as TPC, the antioxidant capacity showed no statistical differences between water regimes. However, values tended to be higher under AWD irrigation compared to flooding, with increases of 5.9 and 16.5% according to ORAC and DPPH assays, respectively. This increase in antioxidant activity could be a clear response of plant acclimatization to water-deficit conditions.

A previous study in pigmented rice reported DPPH values ranging from 2670 to 5810 µmol TE 100 g^−^^1^ in the free fraction and from 340 to 1100 µmol TE 100 g^−^^1^ [54] in the conjugated fraction, which agrees with the values observed in Quila 279101. In contrast, Rocchetti et al. [55] determined an ORAC value of 33.998 µmol TE 100 g^−^^1^ in black rice flour, which is notably higher than the value recorded in the present study. On the other hand, ORAC values between 60 and 106 µmol TE 100 g^−^^1^ in the free fraction and between 207 and 534 µmol TE 100 g^−^^1^ in the combined free/conjugated fraction were described in white rice [56], which are lower than those observed in Zafiro-INIA.

### 3.4. Correlation of Total Phenolic Content (TPC), Antioxidant Capacity, and Color Parameters in Whole-Grain and Polished Rice

The grains of the evaluated genotypes varied in color, being dark purple in Quila 279101, purple in Quila 292008, and white in Zafiro-INIA. These variations in grain color, whether whole or polished, are associated with phenolic content and antioxidant activity. Polished rice is relatively light in color because part of the bran, which contains phenolic compounds of high antioxidant activity, is removed in processing [6,21].

Color parameters, including lightness (L*), redness (a*), and yellowness (b*), serve as robust indicators of the bioactive components in pigmented rice [14]. In this study, significant positive correlations between phenolic content and antioxidant capacity were observed in both polished and whole grains (Figure 2 and Figure 3), which is consistent with the findings reported by Shen et al. [57]. These traits showed a negative correlation with L* and h* in both polished and whole grains, coinciding with the results reported by Shao et al. [13] for insoluble-free, soluble-conjugated, and insoluble-bound fractions. The h* value, which considers both a* and b* values, was initially considered a potentially better indicator of color than a single-color attribute like L* [58]. However, our findings revealed a strong association between TPC, ORAC, and DPPH values and L*. Shen et al. [57] noted a negative correlation of TPC and antioxidant capacity with b* and c*. We detected a similar relationship for c* in whole grains. However, a moderately positive correlation of TPC and antioxidant capacity with b* was found in polished grains. The color and tone of rice grain pericarp are associated with TPC [57,58].

### 3.5. Relative Mineral Composition

The distribution of elements in rice grain occurs from the outer to the inner layer, i.e., husk > bran > whole rice > polished rice [59]. No clear separation was observed between pigmented and white genotypes in whole grains, suggesting no significant differences in element content between the different types of grains. However, Chen et al. [60] reported that black rice had higher concentrations of Mg, Ca, Fe, Mn, and Cu compared to white rice. Under stress conditions, specific enzymes involved in free radical scavenging, which use Fe, Zn, or Cu as electron acceptors in antioxidant processes, are activated [61]. This activation could enhance the uptake of these elements [60]. Furthermore, drought stress reduces the uptake of elements such as K, Ca, Mg, Mn, and B [62]. Similarly, rice exposed to salt stress conditions shows an increase in Fe and Zn and a decrease in Ca levels, as reported by Zhang et al. [59]. In rice, the high variability of grain Fe content associated with the degree of milling, genotypes, growing conditions, and fertilization was reported [63].

In polished grains, AWD irrigation increased the relative content of Co, Ni, Cu, and Zn while causing a decrease in Mn, P, and Si levels. Aerobic conditions enhance soil Zn availability [64], potentially explaining the elevated Zn content observed under AWD conditions. In addition, it should be noted that Zn content was linked to Cu assimilation [60], and Cu was associated with mitigating the effects of drought stress [62]. Interestingly, despite the decrease in Si levels observed in polished grains under water-deficient conditions, it is widely accepted that Si plays an essential role in enhancing drought resistance in crops, including rice [63].

Soil nutrients directly affect plant mineral content [38], and their uptake depends on genotype, environmental conditions, and agronomic practices [64]. The few differences observed in the relative mineral content suggest an effect of the genotype rather than of the environment.

### 3.6. Principal Component Analyses

In black genotypes, clustering was most evident in whole grains, tending to separate according to growing site. The analysis indicates that the environment has a significant impact on grain quality. This is further reinforced by the fact that phenolic compounds and their biological activity mainly characterize the Parral group. At the same time, the mineral composition of Mg, Si, Na, P, K, and Ca largely determines the San Carlos group.

Polished grains exhibited a trend towards genotype clustering, particularly in Quila 292008, primarily attributed to its significantly higher percentage of white belly. On the other hand, Quila 279101-AWD from both cultivation sites were grouped due to their correlation with the concentrations of phenolic compounds, including QCT, AGNd*, Vad*, and VA, along with the TPC and antioxidant activity. The b* color parameter in black genotypes indicated high bioactive compound concentration [14] in polished grain. Meanwhile, the Zafiro-INIA cultivar was consistently associated with transparency and chalkiness, which in turn are related to grain color [4,28,29]. The clear differences between genotypes are mainly responsible for the variations in the content of bioactive compounds within the grains. In addition, metabolite and mineral contents and other grain quality characteristics depend on a combination of genetic factors and environmental conditions [10,24].

## 4. Materials and Methods

### 4.1. Plant Material, Trial Design, and Crop Management

**Figure 7:**
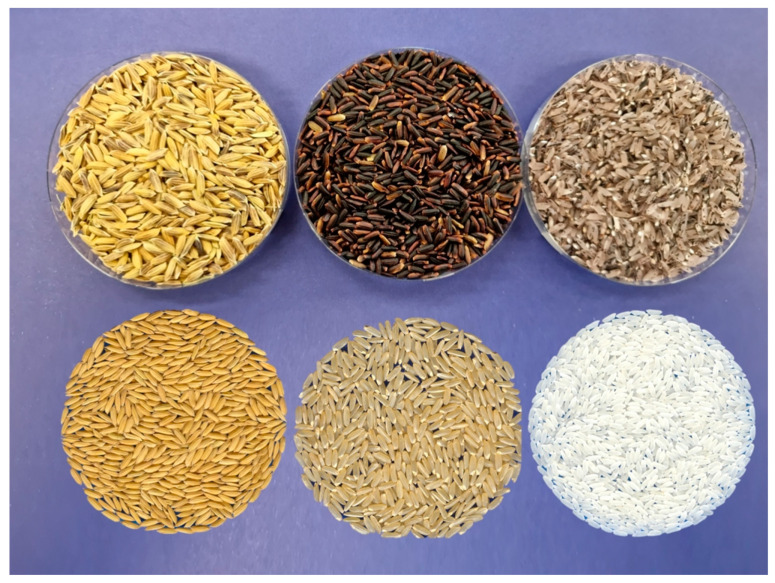
Rice grain appearance of the black rice genotype Quila 292008 (**top**) and the white rice cultivar Zafiro-INIA (**bottom**). On the left, paddy rice; in the center, whole grain; and on the right, polished grain.

**Figure 8:**
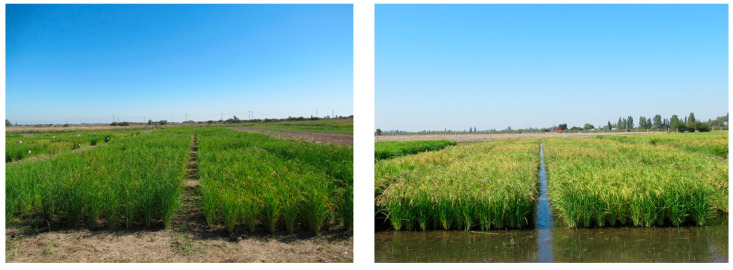
Rice trials in Parral in the 2021 season using different irrigation regimes. (**Left**): alternate wetting and drying, and (**Right**): flooding.

Two black rice genotypes, advanced lines Quila 279101 and Quila 292008, from the RBP of INIA, which were selected based on their performance in a preliminary study with 286 lines grown under water-deficit conditions, and the white rice cultivar Zafiro-INIA were evaluated (Figure 7). The latter represents approximately 70% of the cultivated area of rice in Chile. The trials were established during 2021 in the experimental rice fields of INIA located in San Carlos (36°25′49″ S, 72°0′25″ W; 161 m.a.s.l.) and in Parral (36°04′37″ S; 72°00′13″ O; 142 m.a.s.l.) in the Ñuble and Maule regions, respectively. The germplasm was evaluated under traditional cultivation (flooding) and alternating wetting and drying (AWD) irrigation (Figure 8). The trials were established in the first week of October in both locations. In San Carlos, the length of the growing cycle was 193 (on average) and 186 days under AWD and flooding, respectively. In Parral, AWD and flooding had an average cycle of 178 days.

The genotypes were arranged in a complete randomized block design with three replicates. Each plot was composed of seven rows. The plot was 3 m long and 2.1 m wide, with a row spacing of 0.3 m. Before the establishment of the trials, conventional soil management was conducted, including chemical fallowing using glyphosate (2 L ha^−^^1^), leveling, and soil preparation. Soil was prepared during the dry season to eliminate weeds and improve quality. A shallow plowing and a transverse harrowing were carried out. Contour lines with a height difference of 10 cm were then drawn to form the parapets [3].

The base application of NPK fertilizer was conducted using urea (CH_4_N_2_O) 22 Kg ha^−^^1^, triple superphosphate (Ca(H_2_PO_4_)_2_·H_2_O) 130 Kg ha^−^^1^, and muriate of potassium (KCl: NaCl) 150 Kg ha^−^^1^. Irrigation was initiated one day after sowing, and subsequent irrigation was adjusted based on plant emergence and the prescribed water regime. When plants reached 2.5 leaves and until grain filling was complete, plants under traditional growing conditions remained flooded throughout the growing cycle, while plants in the AWD trial were irrigated every seven days at field capacity. Nitrogen was applied in three more doses: 66 Kg ha^−^^1^ of urea at 2.5 leaves, 88 Kg ha^−^^1^ at tillering, and 44 Kg ha^−^^1^ at panicle initiation. Weed control was performed using Ricer^®^ (210 cc ha^−^^1^) and Clincher^®^ (1.5 L ha^−^^1^) to control narrow-leafed weeds and Bentax (2.5 L ha^−^^1^) and MCPA (1 L ha^−^^1^) to control wide-leafed weeds.

### 4.2. Appearance and Sensorial Quality Traits

The percentage of polished whole grain (WG) was obtained from one hundred clean grains using a test mill (MT, Suzuki Co., Shizuoka, Japan). The weights of one thousand grains of polished rice (TGW-Po) and of paddy rice (TGW-Pa) were determined using an analytical balance. The dimensions of polished grains (length and width) were obtained from grain images with GrainScan 1.0.4 software (CSIRO, Canberra, Australia); subsequently, the length/width ratio (L/W) was calculated. Chalkiness and transparency were assessed with an MM1D milling meter (Satake, Hiroshima, Japan). The percentage of white belly (WB) was calculated as the sum of the values corresponding to grades 1 to 5, which provided the overall percentage of white belly in the sample. The mean degree of dispersion (GDD) was determined from ten representative whole grains incubated for 23 h at 30 °C in 1.7% KOH solution and a subsequent measurement of the gelatinization temperature.

### 4.3. Nutritional Grain Quality Traits

For identification and quantification of anthocyanins and phenolic compounds, as well as for the evaluation of antioxidant capacity, three biological samples (replicates) of each genotype, location, and water condition were used for polished grains, while a composite sample of the three replicates of each genotype, location, and water condition was used for whole grains. In all cases, three technical replicates were evaluated.

### 4.4. Identification and Quantification of Anthocyanins, Flavonols, and Other Phenolic Compounds

Whole and polished grains were ground using an IKA A10 mill (IKA^®^-Werke GmbH & CO., Staufen im Breisgau, Germany). Chemical extraction was performed according to López-Belchí [65] with some modifications. Samples of 0.5 g of the obtained rice powder, 430 nm particle size, were used for metabolite extraction with a 25:24:1 organic solvent mixture (methanol: water: formic acid). Sample mixtures were placed in ultrasound for 1 h, kept at −20 °C overnight, and then ultrasonicated for 1 h.

The resulting extracts were centrifuged (12,000 rpm, 5 min), and the supernatant was collected, filtered through a 0.22 μm PVDF membrane (Millex V13, Millipore, Burlington, MA, USA), and stored at −20 °C until use. Anthocyanins, flavonols, and other phenolic compounds were identified and quantified by chromatography using a Hitachi primaide HPLC-DAD equipped with a diode array detector (Hitachi technologies, Merck, Darmstadt, Germany). The chromatographic system was fitted with a Kromasil C18 column (250 × 4.6 mm, particle size 5 µm) (Nouryon AB., Göteborg, Sweden) to separate metabolites. Chromatographic separation was performed with 1% formic acid and acetonitrile as mobile phases and a flow rate of 1 mL min^−^^1^ [66]. Chromatograms were recorded at 280, 320, 360, and 520 nm. Standards were used to quantify vanillin, vanillic acid, and p-hydroxybenzoic acid at 280 nm; chlorogenic acid, caffeic acid, and coumaric acid at 320 nm; quercetin hydrate and quercetin 3-*O*-glucoside at 360 nm; and cyanidin 3-*O*-glucoside at 520 nm (Sigma-Aldrich, St. Louis, MO, USA). All solvents used in the extractions were of analytical grade and were obtained from Merck (Darmstadt, Germany). Analyses were performed in triplicate, and the results were expressed as mg g^−^^1^ DW.

### 4.5. Total Phenolic Content (TPC)

TPC was determined using the Folin–Ciocalteu assay with modifications for microscale adaptation as described by Gu et al. [67]. Amounts of 25 μL of 0.5 N Folin–Ciocalteu reagent; 25 μL of the sample, blank or standard; and 200 μL of distilled water were added to each well of the microplate. The plate was then shaken for 30 s and incubated for 5 min at 25 °C in the dark. Finally, 25 μL of 10% Na_2_CO_3_ was added, and the absorbance of the samples was measured at 765 nm in a Synergy H1 multimodal hybrid microplate reader (Biotek, Winooski, VT, USA). Results were expressed as mg gallic acid equivalents per 100 g of sample (mg GAE 100 g^−^^1^ sample).

### 4.6. Antioxidant Activity

The antioxidant capacity of the extracts was evaluated with DPPH (2,2-diphenyl-1-picrylhydrazyl) and ORAC (oxygen radical absorbance capacity) assays. The chemicals, including Trolox, fluorescein, and 2,2-azobis-2-amidinopropane dihydrochloride (AAPH), were from Sigma-Aldrich (Sigma-Aldrich, USA). In the first method, the antioxidant activity was determined by measuring the scavenging of the DPPH radical. For this purpose, 25 µL of the extract was mixed with 200 µL of a DPPH solution and incubated in darkness for 1 h. Subsequently, the absorbance was recorded at 515 nm, and the results were expressed as µmol Trolox equivalents per 100 g of rice powder weight (µmol TE 100 g^−^^1^) [67,68].

The ORAC assay was adapted to the microscale according to the method described by Noriega et al. [66] with modifications. Black 96-microwell plates (Nunc, Roskilde, Denmark) and a Synergy H1 multimodal hybrid microplate reader (BioteK, Winooski, VT, USA) were used. In the first microplate columns, 25 μL of Trolox standard was added at 70, 60, 40, 20, 10, and 5 μM. In the following columns of the microplate, 25 μL of sample diluted in 75 mM phosphate buffer pH 7.4 and 25 μL of blank were added. An amount of 150 μL of fluorescein solution was added to each well, and the plate was incubated at 37 °C for 30 min. Then, 25 μL of AAPH was added to each well. Fluorescence reading was performed every 1 min for 60 min with excitation and emission wavelengths of 485 and 520 nm at 37 °C. The calculation of the antioxidant capacity was obtained by the difference of the area under the curve (AUC) between the sample and the blank. Three replicates per Trolox standard, sample, and blank were performed, and the results were expressed as μmol Trolox 100 g^−^^1^ dry weight (µmol TE 100 g^−^^1^) [65].

### 4.7. Grain Color

The grains were placed in a Petri dish, and color parameters CIEL*a* b*, C* (color saturation), and H (hue angle, tan^−^^1^ (b/a)) of grains were measured with a PCECSM 2 colorimeter (Hunter Lab^®^, Murnau am Staffelsee, Germany). Three measurements were taken for each sample.

### 4.8. Relative Mineral Composition of Grain

The method of Cardoso et al. [69] with modifications was used. Spectral signatures of elements in rice grain samples were obtained using a micro-X-ray fluorescence spectrometer (µ-XRF) (M4 Tornado Plus^TM^, Bruker, Bremen, Germany). A total of 48 rice samples (12 whole grains and 36 polished grains) were analyzed, with six technical replicates per sample. Milled and sieved grain samples were placed in 96-well flat-bottom microplates. A stainless-steel rod was used to compact the sample in the well. For the measurements, the X-ray was set at 50 kV and 30 μA with a spot size of 160 μm and an exposition time of 20 ms per spot. Photons were collected with an energy-dispersive silicon drift detector and under atmospheric pressure. Data were expressed as kiloelectronvolts (KeV). The multipoint measurement tool in the Bruker Esprit^TM^ 1.2 software was used for measurements and data processing.

### 4.9. Statistical Analysis

The statistical analysis of grain quality traits was performed by analysis of variance (ANOVA), in which genotypes, location, and water regime were considered fixed factors, and replications were random factors. The least significant difference (LSD) test with a 95% confidence level was used to compare means. Correlation analysis and principal component analysis (PCA) were also performed. These analyses were accomplished in Rstudio version 4.2.1 [70]. The relative mineral composition of the grains was normalized to the 0–1 scale for heat map analysis. Hierarchical clustering was implemented using the Euclidean distance method. The analysis was performed using the Morpheus tool (https://software.broadinstitute.org/morpheus/ (accessed on 12 September 2023)).

## 5. Conclusions

This study evaluated the effect of genotype, location, and water regimen on rice quality traits. The genotypes under study behaved differently. The commercial white rice cultivar exhibited significantly higher grain weight, chalkiness, and transparency than the black rice genotypes and had a higher whole-grain yield. On the other hand, the black rice genotypes showed a higher percentage of white belly. The phenolic compound profiles were markedly different, with cyanidin-3-*O*-glucoside predominating in the pigmented grain and chlorogenic acid in the white grain. In addition, black rice grains showed a significantly higher concentration of phenolic compounds, which in turn resulted in a significantly higher antioxidant capacity.

Water regimen and location influenced the grain quality of rice. Under flooded conditions, heavier grains were obtained, with a significantly higher presence of white belly. Although several grain quality traits were negatively affected by AWD irrigation, the concentration of phenolic compounds tended to increase under this water regimen, probably due to plant acclimatization to water stress. However, no significant differences were found in total phenolic content or antioxidant activity. Furthermore, there were differences in grain relative mineral content due to water regime, especially in polished grains. Higher grain weight was obtained in Parral, where the concentration of phenolic compounds showed an upward tendency. Nevertheless, there were no differences in total phenolic content or antioxidant activity between growing sites. In addition, higher whole-grain yields were obtained in San Carlos, while the relative mineral content also varied between locations.

As expected, black rice genotypes showed marked differences in phenolic content and antioxidant activity. However, no differences were observed in total phenolic content or antioxidant activity due to water regime or location, suggesting that the production and accumulation of these compounds in the grain are strongly genetically determined. This study provides insights into the relationship between genotype, growing location, and water regime on rice quality. Nevertheless, further research is needed to understand the environmental impact on rice grain quality.

## Figures and Tables

**Table 1:** Analysis of variance of grain-weight and grain-size traits of three rice genotypes grown in different locations and under different water regimes.

	TGW-Pa		TGW-Po		Length		Width		L/W	
	(g)		(g)		(mm)		(mm)			
**Genotype (G)**										
Quila 279101	27.83	±1.20 ^b^	18.54	±0.81 ^b^	6.12	±0.13 ^a^	2.40	±0.04 ^a^	2.55	±0.06 ^ab^
Quila 292008	24.90	±1.35 ^c^	17.54	±0.89 ^c^	6.07	±0.14 ^a^	2.30	±0.04 ^b^	2.64	±0.07 ^a^
Zafiro	34.48	±1.45 ^a^	24.54	±1.01 ^a^	6.07	±0.17 ^a^	2.47	±0.04 ^a^	2.45	±0.07 ^b^
**Location (L)**										
San Carlos	28.34	±1.09 ^b^	19.99	±0.77 ^b^	6.12	±0.13 ^a^	2.40	±0.03 ^a^	2.56	±0.06 ^a^
Parral	29.79	±1.04 ^a^	20.42	±0.72 ^a^	6.05	±0.13 ^a^	2.38	±0.03 ^a^	2.54	±0.05 ^a^
**Water regime (W)**										
Flooding	29.98	±0.95 ^a^	20.48	±0.71 ^a^	6.18	±0.10 ^a^	2.44	±0.03 ^a^	2.54	±0.05 ^a^
AWD	28.16	±1.09 ^b^	19.93	±0.77 ^b^	5.99	±0.15 ^a^	2.34	±0.03 ^b^	2.56	±0.06 ^a^
***p*-value**										
G × L	0.1950		0.3438		**0.0038**		0.1323		**0.0007**	
G × W	0.2917		0.3823		**0.0058**		**0.0454**		**0.0005**	
L × W	0.5028		**0.0073**		0.5192		0.2501		0.1374	
G × L × W	**0.0337**		0.4700		**0.0038**		0.1703		**0.0020**	

TGW-Pa: thousand-grain weight of paddy rice, TGW-Po: thousand-grain weight of polished rice, L/W: ratio length/wide of polished grain. AWD: alternate wetting and drying. Data are mean values ± standard error (n = 3). Means with different letters are statistically different according to the LSD Fisher test. Bold *p*-values indicate significant interactions between factors (*p* ≤ 0.05). There were no statistical differences in grain length between genotypes, locations, or water regimes (Table 1). However, grain width was significantly smaller in Quila 292008, with no differences between the white rice cultivar and the other black rice genotype, being 4.1% wider in plants under flooding. Regarding the grain length/width ratio, significant differences were found between Quila 292008 and Zafiro-INIA, but no differences were detected between Quila 279101 and Quila 292008 or Zafiro-INIA.

**Table 2:** Analysis of variance of grain quality traits of three rice genotypes grown in different locations and under different water regimes.

	WG		CHA		TRAN		WB		ADD	
	(%)						(%)			
**Genotype (G)**										
Quila 279101	41.07	±4.59 ^b^	12.32	±3.09 ^c^	0.56	±0.37 ^c^	15.67	±3.77 ^b^	6.48	±0.14 ^a^
Quila 292008	52.53	±3.67 ^a^	15.61	±3.04 ^b^	0.80	±0.37 ^b^	23.83	±3.51 ^a^	6.17	±0.16 ^ab^
Zafiro	56.19	±2.76 ^a^	36.53	±3.44 ^a^	3.50	±0.41 ^a^	7.17	±1.77 ^c^	5.78	±0.19 ^b^
**Location (L)**										
San Carlos	52.97	±3.42 ^a^	21.82	±2.59 ^a^	1.57	±0.32 ^a^	17.44	±2.88 ^a^	6.05	±0.15 ^a^
Parral	46.88	±3.94 ^b^	21.16	±2.45 ^a^	1.66	±0.30 ^a^	13.67	±2.87 ^a^	6.24	±0.14 ^a^
**Water regime (W)**										
Flooding	36.96	±3.42 ^b^	22.47	±2.61 ^a^	1.67	±0.33 ^a^	21.67	±3.00 ^a^	6.34	±0.13 ^a^
AWD	62.89	±0.88 ^a^	20.50	±2.51 ^b^	1.56	±0.31 ^a^	9.44	±1.58 ^b^	5.95	±0.16 ^b^
***p*-value**										
G × L	0.8333		0.7814		0.2574		0.4935		0.7068	
G × W	**0.0121**		0.6876		0.2066		**0.0030**		0.1177	
L × W	0.0612		0.8629		0.2072		0.8410		0.4370	
G × L × W	0.7243		0.1621		0.0819		0.1370		0.2913	

WG: whole-grain yield, CHA: chalkiness, TRAN: transparency, WB: white-belly rate, and ADD: average degree of dispersion of alkaline reaction. AWD: alternate wetting and drying. Data are mean values ± standard error (n = 3). Means with different letters are statistically different according to the LSD Fisher test. Bold *p*-values indicate significant interactions between factors (*p* ≤ 0.05).

**Table 3:** Analysis of variance of grain polyphenolic content and antioxidant capacity of three rice genotypes grown in different locations and under different water regimes.

	TPC		ORAC		DPPH	
	(mg GAE 100 g^−1^)		(µmol TE 100 g^−1^)		(µmol TE 100 g^−1^)	
**Genotype (G)**						
Quila 279101	98.37	±13.15 ^a^	2605.63	±299.37 ^a^	510.38	±96.27 ^a^
Quila 292008	51.14	±12.87 ^b^	1343.05	±259.12 ^b^	290.87	±94.08 ^b^
Zafiro	26.05	±13.43 ^c^	728.34	±268.61 ^c^	58.92	±82.81 ^c^
**Location (L)**						
Parral	58.57	±10.00 ^a^	1554.18	±227.18 ^a^	260.09	±72.70 ^a^
San Carlos	58.47	±9.63 ^a^	1563.83	±207.92 ^a^	313.35	±70.61 ^a^
**Water regime (W)**						
Flooding	48.22	±7.05 ^a^	1511.25	±223.01 ^a^	260.87	±70.50 ^a^
AWD	68.82	±11.57 ^a^	1606.76	±226.22 ^a^	312.57	±72.62 ^a^
***p*-value**						
G × L	0.3649		0.4454		0.7376	
G × W	0.8585		0.6144		0.1910	
L × W	0.3072		0.7362		0.3225	
G × L × W	**0.0099**		**0.0413**		**0.0314**	

TPC: total polyphenolic content, ORAC: oxygen radical absorbance capacity assay, DPPH: 2,2-diphenyl-1-picrylhydrazyl assay, GAE: gallic acid equivalent, TE: Trolox equivalent, AWD: alternate wetting and drying. Data are mean values ± standard error (n = 3). Means with different letters are statistically different according to the LSD Fisher test. Bold *p*-values indicate significant interactions between factors (*p* ≤ 0.05).

## Data Availability

All data were generated during the study. They are not publicly available, but can be accessed through the following GoogleDrive link: https://docs.google.com/spreadsheets/d/17nidj8qAv5MNQJHvrhau-Y92K0IFriod/edit?usp=sharing&ouid=108551625874148646272&rtpof=true&sd=true (accessed on 5 April 2023).

## Supplementary Materials

The following supporting information can be downloaded at: https://www.mdpi.com/article/10.3390/plants12244091/s1, Table S1: Analysis of variance of grain yield of three rice genotypes grown in different locations and under different water regimes. Table S2: Mineral concentration in polished rice of three genotypes (Quila 279101, Quila 292008, and Zafiro-INIA) grown in two locations (San Carlos and Parral) and under two water regimes (flooding and AWD). Table S3: Concentration of phenolic compounds (mg g^−1^) in extracts of whole-grain composite samples of three rice genotypes (Quila 279101, Quila 292008, and Zafiro-INIA) grown in two locations (San Carlos and Parral) and under two water regimes (flooding and alternate wetting and drying). Table S4: Concentration of phenolic compounds in polished grain extracts of three rice genotypes (Quila 279101, Quila 292008, and Zafiro-INIA) grown in two locations (San Carlos and Parral) and under two water regimes (flooding and alternate wetting and drying). Figure S1: Agrometeorological data of air temperature (maximum, minimum, and average) and accumulated rainfall recorded in San Carlos in the 2020–2021 season. Figure S2: Agrometeorological data of air temperature (maximum, minimum, and average) and accumulated rainfall recorded in Parral in the 2020–2021 season. Figure S3: Chromatogram of a polished grain sample of the Quila 279101 genotype measured in HPLC at 320 nm. Figure S4: Chromatogram of a whole-grain sample of the cultivar Zafiro-INIA measured in HPLC at 320 nm.
