# Supplementary material for: Assessing Grain Quality Changes in White and Black Rice under Water Deficit
Source: Plants (Basel). 2023 Dec 7;12(24):4091. doi: 10.3390/plants12244091 (PMC10748231; doi:10.3390/plants12244091)
Supplement: Supplementary file 1 [file plants-12-04091-s001.zip › plants-2695388-supplementary.pdf]

Supplementary Data

Table S1. Analysis of variance of grain yield of three rice genotypes grown in different locations and water regimes.

	GY (ton ha^{-1})
**Genotype G)**	
Quila 279101	5.16 ± 0.80 b
Quila 292008	3.15 ± 1.01 c
Zafiro	6.59 ± 1.02 a
**Location (L)**	
San Carlos	5.03 ± 0.75 a
Parral	4.91 ± 0.74 a
**Water regime (W)**	
Flooding	7.35 ± 0.57 a
AWD	2.58 ± 0.32 b
**p-value**	
G x L	**0.0031**
G x W	**0.0001**
L x W	**0.0001**
G x L x W	**0.0004**

GY: grain yield, AWD: Alternate wetting and drying. Data are mean values ± standard error (n = 3). Means with different letters are statistically different according to the LSD Fischer test (p ≤ 0.05).

Table S2. Mineral concentration in polished rice of three genotypes (Quila 279101, Quila 292008, and Zafiro-INIA) grown in two locations (San Carlos and Parral) and under two water regimes (flooding and AWD).

	**Na** (KeV)	**Mg** (KeV)	**Si** (KeV)	**P** (KeV)	**S** (KeV)	**K** (KeV)	**Ca** (KeV)	**Mn** (KeV)	**Fe** (KeV)	**Co** (KeV)	**Ni** (KeV)	**Cu** (KeV)	**Zn** (KeV)
Genotype (G)													
Quila 279101	36.62 ab	30.45	44.84	39.52 a	33.35	408.22 a	50.96 a	291.46 a	343.26	384.04	477.92	608.87	871.27
Quila 292008	38.72 a	29.42	45.33	38.75 b	35.85	368.08 ab	44.63 ab	284.38 ab	391.64	373.62	466.83	596.44	838.97
Zafiro	35.79 b	30.01	43.93	30.72 b	35.54	294.91 b	37.74 b	266.57 b	335.77	384.33	480.97	607.16	876.02
Location (L)													
Parral	36.92	30.21	45	37.1	36.06	370.53	45.36	283.66	369.49	374.31	469.75	600.97	844.54
San Carlos	37.16	29.71	44.4	35.56	33.76	343.60	43.53	277.94	344.29	387.02	480.73	607.34	879.64
Water regime (W)													
Flooding	36.78	30.06	44.7	36.06	32.94	357.87	45.09	287.97	330.55	378.24	469.19	598.48	836.70 b
AWD	37.30	29.86	44.7	36.6	36.89	356.26	43.79	273.63	383.22	383.09	481.29	609.83	887.47 a
p-value													
G x L	0.952	0.582	0.943	0.896	0.558	0.9505	0.424	0.1142	0.582	0.6235	0.385	0.5162	0.7658
G x W	0.502	0.325	0.859	0.573	0.439	0.8045	0.844	0.4407	0.105	0.1481	0.228	0.3318	0.3356

L x W	0.502	0.098	0.84	0.559	0.331	0.6048	0.600	0.058	0.403	0.3318	0.772	0.6584	0.9981
G x L x W	0.444	0.196	0.918	0.388	0.252	0.3867	0.165	0.8092	0.782	0.3618	0.216	0.7155	0.9646

KeV: Kiloelectron Volt. Means with different letters are statistically different according to the LSD Fischer test ($p \leq 0.05$). AWD: Alternate wetting and drying. Data are mean values. n = 3.

Table S3. Concentration of phenolic compounds (mg g^{-1}) in extracts of whole grain composite samples of three rice genotypes (Quila 279101, Quila 292008, and Zafiro-INIA) grown in two locations (San Carlos and Parral) and under two water regimes (flooding and alternate wetting and drying).

Genotype	Location	Condition	VA	VAd*	PHBA	VNL	CGA	CA	AGNd*	QCT	C3G	Sum phenolic compound
Quila 279101	PA	F	0.096	0.162	nd	nd	0.046	0.018	0.169	0.090	3.066	3.646
Quila 279101	PA	AWD	0.110	0.191	nd	nd	0.029	0.065	0.200	0.106	4.141	4.841
Quila 279101	SC	F	0.101	0.139	nd	nd	0.061	0.020	0.148	0.073	2.216	2.758
Quila 279101	SC	AWD	0.087	0.180	nd	nd	0.063	0.020	0.116	0.065	0.865	1.396
Quila 292008	PA	F	0.068	0.160	nd	0.025	0.017	0.058	0.137	0.086	1.514	2.065
Quila 292008	PA	AWD	0.077	0.222	nd	0.016	0.027	0.062	0.159	0.087	3.708	4.358
Quila 292008	SC	F	0.051	0.141	nd	0.013	0.072	0.021	0.092	0.055	0.524	0.971
Quila 292008	SC	AWD	0.038	0.122	nd	0.029	0.062	0.018	0.074	0.035	0.168	0.546
Zafiro-INIA	PA	F	0.013	nd	0.034	nd	0.036	0.008	0.008	nd	nd	0.099
Zafiro-INIA	PA	AWD	0.014	nd	0.003	nd	0.042	0.010	0.021	nd	nd	0.089
Zafiro-INIA	SC	F	0.014	nd	0.045	nd	0.049	0.017	0.010	nd	nd	0.135
Zafiro-INIA	SC	AWD	0.012	nd	0.018	nd	0.039	0.009	0.018	nd	nd	0.096

nd: non-detected. AWD: Alternate wetting and drying. F: Flooding. PA: Parral. SC: San Carlos. Quila 219101 and Quila 292008; Black rice. Zafiro-INIA; White rice. VA: Vanillic acid, VAd*: Vanillic acid derivative, PHBA: p-hydroxybenzoic acid, VNL: Vanillin, CGA: Chlorogenic acid, CA: Caffeic acid, AGNd*: Apigenin derivative, QCT: Quercetin, C3G: Cyanidin 3-*O*-Glucoside.

Table S4. Concentration of phenolic compounds in polished grain extracts of three rice genotypes (Quila 279101, Quila 292008, and Zafiro-INIA) grown in two locations (San Carlos and Parral) and under two water regimes (flooding and alternate wetting and drying).

Genotype	Location	Condition	VA (mg g^{-1})	Vad* (mg g^{-1})	CGA (mg g^{-1})	CA (mg g^{-1})	AGNd* (mg g^{-1})	QCT (mg g^{-1})	C3G (mg g^{-1})	Sum phenolic compound (mg g^{-1})
Quila 279101	PA	F	0.048 ± 0.004	0.061 ± 0.013	0.016 ± 0.007	0.013 ± 0.002	0.066 ± 0.015	0.021 ± 0.008	0.047 ± 0.027	0.272 ± 0.069
Quila 279101	PA	AWD	0.046 ± 0.007	0.050 ± 0.011	0.028 ± 0.007	0.010 ± 0.002	0.064 ± 0.015	0.018 ± 0.005	0.071 ± 0.005	0.287 ± 0.041
Quila 279101	SC	F	0.037 ± 0.005	0.045 ± 0.012	0.018 ± 0.002	0.006 ± 0.001	0.048 ± 0.009	0.013 ± 0.004	0.013 ± 0.006	0.181 ± 0.038
Quila 279101	SC	AWD	0.045 ± 0.002	0.090 ± 0.007	0.015 ± 0.006	0.010 ± 0.003	0.086 ± 0.006	0.036 ± 0.005	0.123 ± 0.021	0.405 ± 0.044
Quila 292008	PA	F	0.014 ± 0.001	0.025 ± 0.002	0.005 ± 0.004	0.021 ± 0.001	0.023 ± 0.003	0.007 ± 0.001	1.069 ± 0.086	1.165 ± 0.089
Quila 292008	PA	AWD	0.027 ± 0.003	0.051 ± 0.006	0.035 ± 0.003	0.010 ± 0.002	0.057 ± 0.010	0.019 ± 0.005	0.043 ± 0.012	0.241 ± 0.039
Quila 292008	SC	F	0.016 ± 0.001	0.031 ± 0.04	0.022 ± 0.004	0.021 ± 0.001	0.026 ± 0.001	0.008 ± 0.000	1.129 ± 0.126	1.253 ± 0.124
Quila 292008	SC	AWD	0.010 ± 0.001	0.027 ± 0.003	0.023 ± 0.002	0.010 ± 0.002	0.026 ± 0.002	0.008 ± 0.001	2.189 ± 0.130	2.292 ± 0.131
Zafiro	PA	F	0.012 ± 0.000	nd	nd	0.011 ± 0.002	0.008 ± 0.001	nd	nd	0.031 ± 0.003

Zafiro	PA	AWD	0.011 ± 0.000	nd	0.027 ± 0.002	0.010 ± 0.001	0.007 ± 0.000	nd	nd	0.054 ± 0.001
Zafiro	SC	F	0.013 ± 0.000	nd	nd	0.008 ± 0.001	0.003 ± 0.002	nd	nd	0.024 ± 0.003
Zafiro	SC	AWD	0.009 ± 0.004	nd	nd	0.021 ± 0.007	0.008 ± 0.004	nd	nd	0.038 ± 0.007

nd: non-detected. AWD: Alternative wet and drying. F: Flooding. PA: Parral. SC: Quila 219101 y Quila 292008: Black rice. Zafiro-INIA: White rice. VA: Vanillic acid, VAd*: Vanillic acid derivative, CGA: Chlorogenic acid, CA: Caffeic acid, AGNd*: Apigenin derivative, QCT: Quercetin, C3G: Cyanidin 3-*O*-Glucoside. Data are mean values ± standard error (n = 3).

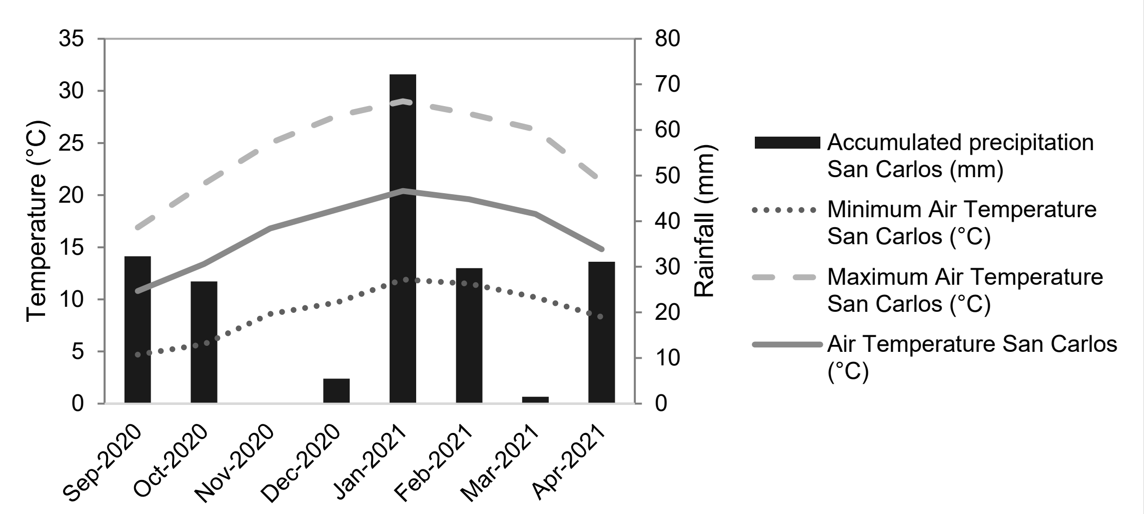

Figure S1. Agrometeorological data of air temperature (maximum, minimum, and average), and accumulated rainfall recorded in San Carlos in the 2020-2021 season.

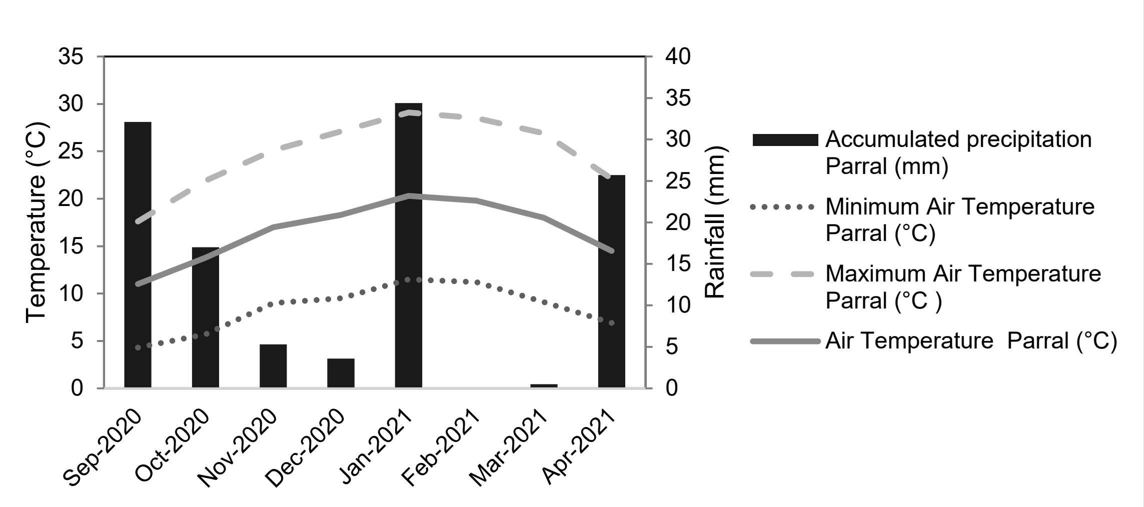

Figure S2. Agrometeorological data of air temperature (maximum, minimum, and average), and accumulated rainfall recorded in Parral in the 2020-2021 season.

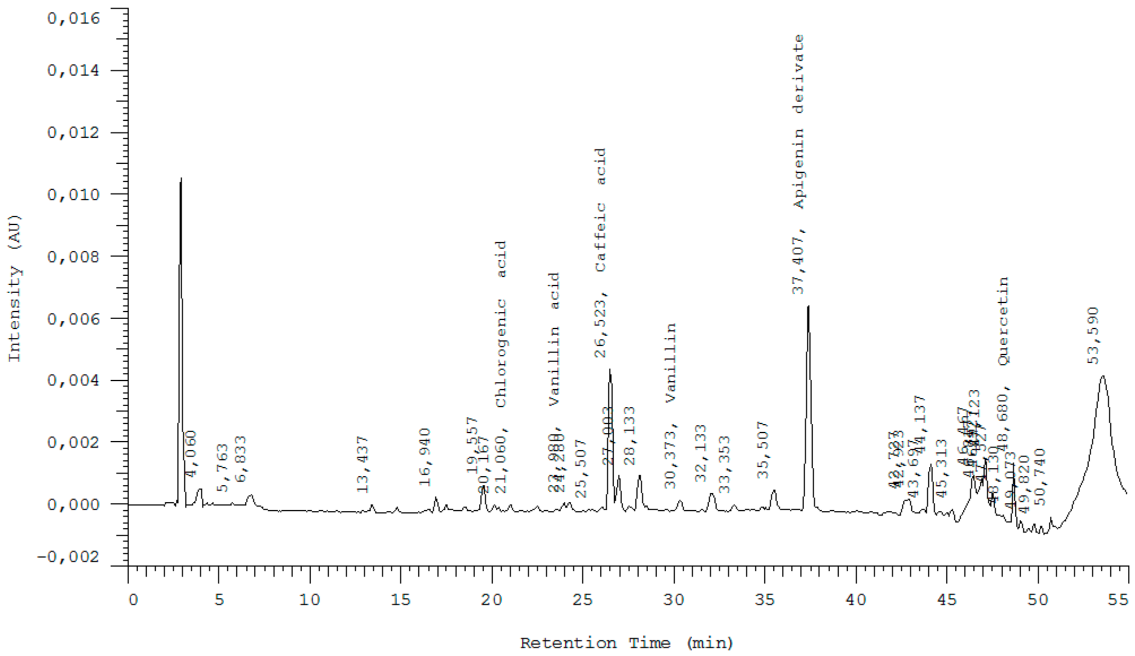

Figure S3. Chromatogram of a polished grain sample of the Quila 279101 genotype measured in HPLC at 320 nm.

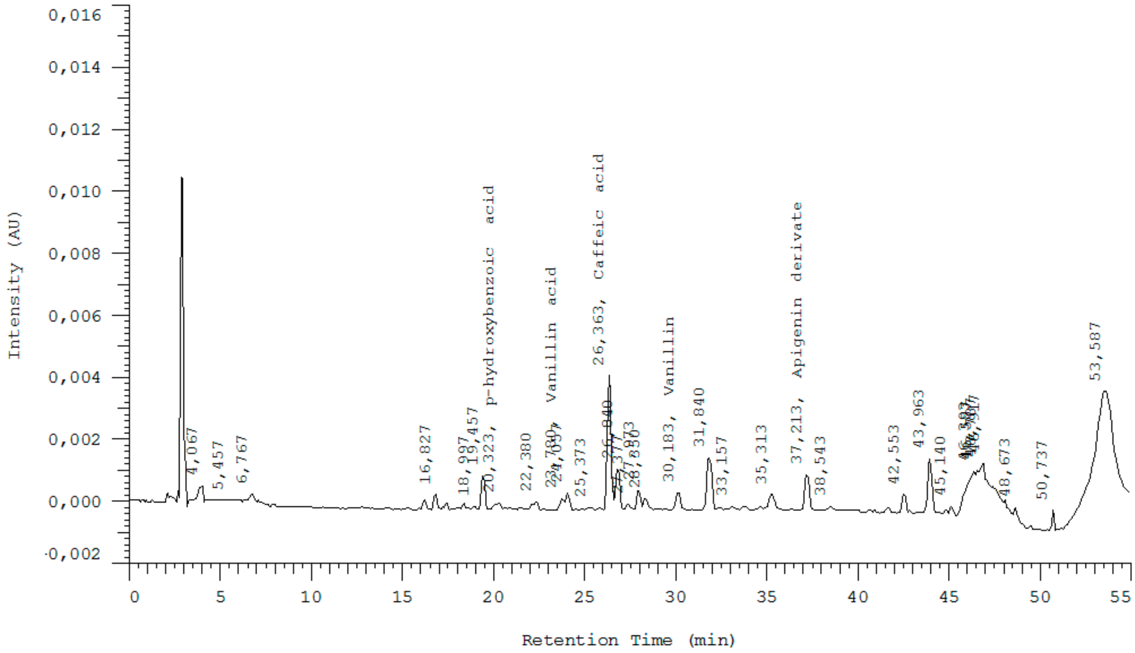

Figure S4. Chromatogram of a whole grain sample of the cultivar Zafiro-INIA measured in HPLC at 320 nm.
